# Identification of Key Players Involved in CoCl_2_ Hypoxia Induced Pulmonary Artery Hypertension *in vitro*

**DOI:** 10.3389/fgene.2020.00232

**Published:** 2020-04-24

**Authors:** Shu Chen, Hui Xu, Fen Hu, Tao Wang

**Affiliations:** ^1^Department of Respiratory and Critical Care Medicine, Tongji Hospital, Tongji Medical College, Huazhong University of Science and Technology, Wuhan, China; ^2^Department of Cardiovascular Surgery, Union Hospital, Tongji Medical College, Huazhong University of Science and Technology, Wuhan, China

**Keywords:** pulmonary artery hypertension, hypoxia, long non-coding RNA, transcriptome sequence, functional enrichment analyses

## Abstract

**Background:**

The proliferation of human pulmonary artery smooth muscle cells (HPASMCs) induced by hypoxia was considered as the main cause of pulmonary arterial hypertension (PAH). This study aimed to explore potential genes and long non-coding RNAs (lncRNAs) involved in the mechanism of hypoxia-induced PAH.

**Methods:**

CoCl_2_ was utilized to induce hypoxia in HPASMCs, and then cell proliferation, apoptosis, and expression of hypoxia-inducible factors (HIF)-1α were determined. Meanwhile, the RNA isolated from CoCl_2_-treated cells and control cells were sequenced and differentially expressed genes/lncRNA (DEGs/DELs) were screened, followed by protein-protein interaction (PPI) construction, functional enrichment analyses, and lncRNA-target prediction. Finally, the expression of key genes and lncRNAs were validated using quantitative real-time PCR and western blotting.

**Results:**

CoCl_2_ treatment could significantly increase the expression of HIF-1α and the proliferation of HPASMCs. A total of 360 DEGs and 57 DELs were identified between CoCl_2_ treated and control cells. Functional enrichment analysis showed that up-regulated DEGs and DELs’ targets, including LDHA, PFKP, and VEGFA, were significantly enriched in biological processes related to hypoxia or oxygen levels, and the downregulated DEGs and DELs’ targets were significantly enriched in extracellular-matrix-related biological processes. In addition, LDHA, PFKP, and VEGFA exhibited a strong relationship with miR-100HG and TSPEAR-AS2 in lncRNA-target network. The protein level of LDHA, PFKP, and VEGFA were all increased.

**Conclusion:**

LDHA, PFKP, VEGFA, and lncRNA miR-100HG and TSPEAR-AS2 probably played crucial roles in the pathogenesis of CoCl_2_ hypoxia-induced-HAP, which might serve as promising therapeutic targets for PAH.

## Introduction

Pulmonary hypertension (PH) is a common complicated disorder characterized by a rest mean pulmonary artery pressure of >25 mm Hg. The World Health Organization (WHO) categorized PH into 5 categories according to common clinical parameters, potential pathology, pathophysiology, etiology, and therapies. Specifically, pulmonary arterial hypertension (PAH) belongs to the WHO Group I characterized by an increased vasomotor tone, vascular resistance from abnormal narrowing, and precapillary vasculature remolding associated with abnormalities of proliferation and apoptosis (Simonneau et al., 2013; Ramchandran et al., 2014; Yun et al., 2016). Although available therapies for PAH are commonly designed to revise the elevated vascular tone and vascular remodeling, the outcomes of these therapies are still not satisfied in clinic (Michelakis et al., 2008). Therefore, a better understanding of the pathology of PAH is necessary to develop some useful therapies to manage this syndrome (Lau et al., 2015).

Hypoxia is one of the most important causes for vascular remodeling during the parthenogenesis of PAH (Montani et al., 2013; Xia et al., 2014). Chronic alveolar hypoxia is likely to promote pulmonary vasoconstriction and vessel remolding (Shimoda et al., 2000; Kuriyama et al., 2014). Hypoxia inducible factors (HIFs) are a class of transcriptional factors that respond to hypoxia (Veith et al., 2016). Among these, *HIF-1*α has been identified to play a critical role in the regulation of PAH (Yue et al., 2013). Roger A et al. have demonstrated that *HIF-1*α is crucial for the hypoxia-induced mitogenic factor induced PH (Johns et al., 2016). Li et al. (2015) have documented that *KLF5* mediates hypoxia-induced vascular remodeling via *HIF-1*α. Meanwhile, inhibiting the expression of HIF-1α alleviates hypoxia-induced PH (Ying et al., 2016). Moreover, *Pyk2* contributes to hypoxia-induced PH via activating *HIF-1*α (Kuniyoshi et al., 2015). Jiang et al. (2015) have also reported that S*UMO-1* can interact with *HIF-1*α to regulate sumoylation of *HIF-1*α responding to hypoxia. Despite these findings, the mechanism of *HIF-1*α in PH is still not clearly understood.

Long non-coding RNAs (lncRNA) are a class of transcriptomes with non-protein coding ability and length ≥200 nt (Tian and Xu, 2015), and have been identified as playing critical roles in the regulation of cell activities, including metabolism, proliferation, apoptosis, metastasis, and invasion (Liu et al., 2016; Sun et al., 2016; Gasri-Plotnitsky et al., 2017; Peng et al., 2017). Recently, Brock et al. (2016) have reported that lncRNA *MEGs* serves as an emerging factor in hypoxia-induced PH. Zhuo et al. (2016) have observed that polymorphism re619586A >G in lncRNA *MALAT1* contributes to PAH susceptibility in Chinese people. Zhang et al. (2016) have identified that lncRNA *BCYRN1* promotes the proliferation and migration of rat airway smooth muscle cells in asthma via elevating the expression of *TRPC1*. All of these findings indicate that lncRNAs also play critical roles in the regulation of PAH, but evidence in this area is still rarely reported.

To further reveal the pathogenesis of PAH, the effect of *HIF-1*α on PAH was identified. Meanwhile, CoCl_2_ treated cells and their corresponding controls were sequenced and analyzed using a bioinformatics method in pulmonary artery smooth muscle cells to further explore the genes and lncRNAs involved in hypoxia-induced PAH pathogenesis, providing some new insights in the understanding and treatment of PAH.

## Materials and Methods

### Ethics Approval and Consent to Participate

This article does not contain any studies with human participants or animals performed by any of the authors.

### Cell Culture and Treatment

In the current study, two cell lines, human pulmonary artery smooth muscle cells (HPASMCs), and human embryonic kidney cells 293T, were used for the following investigations. 293T cell lines were maintained in Dulbecco’s modified eagle medium (DMEM, Gibco, Grand Island, NY, United States) supplemented with 10% of fetal bovine serum (FBS) and Penicillin/Streptomycin at 37°C with 5% CO_2_. HPASMCs were maintained in DMEM supplemented with 15% of FBS at 37°C with 5% CO_2_ (Li et al., 2014).

### CCK-8 Assay

The proliferation of cells treated with different concentrations of CoCl_2_ were determined using a CCK-8 kit (DOJINDO, Japan). Briefly, HPASMCs were seeded in 96-well plate with 2 × 10^4^ for each well overnight. After adherence, cells were separately treated with 50, 100, or 200 μM CoCl_2_ for 24 and 48 h. Then, 10 μl CCK-8 solution was added in each well and the cells were cultured at 37°C for 2 h. Following this, the optical density (OD) of each well at 450 nm was measured using an Enzyme-linked immunoassay instrument (TECAN Infinite M100 PRO, CA, United States). Each treatment was repeated six times, and the mean value of OD was calculated as the final result.

### 5-Bromo-Deoxyuridine (BrdU) Assay

The BrdU assay was performed according to the protocol of the BrdU Cell Proliferation Assay Kit (Frdbio, China). Briefly, BrdU labeling solution was added to each well for 4 h. After cells were fixed and DNA denatured, 100 μl BrdU detection antibody were added under a condition of mild vibrating at room temperature for 1 h. Then, the anti- BrdU HRP conjugated antibody was added to the cultures and incubated for 1 h at room temperature. After three washing steps the bound peroxidase was detected by subsequent substrate reaction. This reaction was stopped by adding 100 μl stop buffer and quantified by measuring the optical density (OD) at a wavelength of 450 nm on a microplate reader (BioTek, United States).

### Apoptosis

Apoptosis for HPASMCs with CoCl_2_ treatment was also determined in the present study. Briefly, HPASMCs were seeded in six-well plate with 4 × 10^5^ for each well overnight followed by the treatment with 200 μM CoCl_2_ and DMSO for 48 h. After that, cells were trypsinized and centrifuged at 1500 rpm for 6 min. The cell sediment was washed with phosphate buffer solution and re-suspended with 1 × Binding Buffer. Then, 100 μl cell-binding buffer was collected, and incubated with 5 μl FITC-Annexin V (BD, United States) and 5 μl PI (50 μg/ml) at room temperature in a dark place. Meanwhile, unstaining, single Annexin-V staining, and single PI staining were also performed to minimize error during these procedures. After incubation, solution was mixed with 400 μl 1 × Binding Buffer and measured using a flow cytometry. Each experiment was performed three times, and the mean value was computed.

### Sequencing and Bioinformatics Analysis

With a confluency of 70∼80%, HPASMCs were treated with 200 μM CoCl_2_ for 48 h, and DMSO were used as the control treatment. Each treatment was performed three times. Then, total RNA of cell samples was isolated and sequenced on the Hiseq4000 sequencing platform (Illumina). Based on the recorded data, lower quality of reads and beads were filtered out, and the clean reads were calculated. Following this, clean reads were mapped to human reference genome 19 using tophat (Trapnell et al., 2009). According to the known genes and lncRNA annotation provided by gencode v24 (Harrow et al., 2012), fpkm and read count of sequencing genes were obtained using stringtie (Pertea et al., 2015), and differentially expressed genes (DEGs) and differentially expressed lncRNAs (DELs) were screened using LIMMA in R (Ritchie et al., 2015) with the thresholds of |log2 Fold change (FC)| >1 and false discovery rate (FDR) <0.05. Based on the Spearman rank correlation coefficient, targets of lncRNAs were screened from DEGs with the thresholds of *P* < 0.05 and coefficient >0.8, and the lncRNA-target regulatory network was visualized using Cytoscape (Shannon et al., 2003). Furthermore, Gene ontology (GO) and Kyoto Encyclopedia of Genes and Genomes (KEGG) enrichment analyses of DEGs, as well as the targets of lncRNAs, were also carried out using clusterprofile package in R (Yu et al., 2012). Finally, protein-protein interactions (PPIs) among DEGs were predicted using STRING (Franceschini et al., 2012), and PPI network was visualized using Cytoscape.

### Quantitative Real-Time PCR (RT-PCR)

Total RNA was isolated using Trizol Regent (Invitrogen, Carlsbad, CA, United States), according to the manufacture’s protocol. Then, RNA concentration was quantified using NanoDrop 2000 (Thermo, Wilmington, DE, United States), and 0.5 μg total RNA was reversed to cDNA using 5 × primeScript RT Master Mix (Takara, Dalian, China), according to its instructions. Subsequently, gene expression was measured using SYBR Premix EX Taq (2 ×) with the following procedure: 50°C for 3 min, 95°C for 3 min, and 40 cycles of 95°C for 10 s and 60°C for 30 s. The relevant target gene expression levels were calculated with the 2-ΔΔCT method (Livak and Schmittgen, 2001). Primers of genes were tabulated in Table 1.

**TABLE 1:** Primers for real-time PCR.

Primers	Forward	Reverse
HIF-1α	5′-GCCCTAACGTGTT ATCTGTCG-3′	5′-TTGCTCCATT CCATTCTGTTC-3′
PFKP	5′-TACTTCATCTACGAG GGCTACCA-3′	5′-AATGATCGTCCCG CCCACTT-3′
VEGFA	5′-GCCTTGCCTTGCT GCTCTAC-3′	5′-CTCGATTGGATGG CAGTAGC-3′
LDHA	5′-GTCAGCAAGAGGGA GAAAGC-3′	5′-TCCAAGCCACGTAG GTCAAG-3′
HIST1H1B	5′-CTGGTGCTTCTG GCTCCTTTAA-3′	5′-CGCCTTCTTCGG AGTCTTCTT-3′
HIST2H4A	5′-ACAACATTCAGG GCATCACCA-3′	5′-CTCCAGGAACACCT TCAGCACA-3′
MIR100HG	5′-ACATTGGTCCAC TTGACACT-3′	5′-CAACTTGATGGCAA ACTCCC-3′
RP11-166D19	5′-TGCCAGTCAGC CTTTGTAGT-3′	5′-AGAGTTTGGGAGC AGAGGGA-3′
AP001065.15	5′-CCAGGCAAGAAC CCAGGATA-3′	5′-ACACTCAGGCAG GCTCCCAC-3′
TSPEAR-AS2	5′-GGACATCACTCGC CTTCAGA-3′	5′-CTAACGAATGAGCT GGTGGG-3′
DYX1C1-CCPG1	5′-CTCGCTCTGTTG GCAGTATTA-3′	5′-TCATTGCTCTT CGTGCCTCA-3′
LINC00951	5′-CAGCCCTTTCCACCT ACTACAT-3′	5′-GCATGTGGGTGTTG GTGAGA-3′
β-actin	5′-GGCCGAGGAC TTTGATTGCA-3′	5′-GGGACTTCCTGTT AACAACGCA-3′

### Western Blotting

Cells were lysed in RIPA lysis buffer (Beyotime, China) coupled with PMSF (Beyotime) at a final concentration of 1 mM, followed by the determination of protein concentration using the BCA Protein Assay Kit (Thermo). Protein from each sample was separated by 10% SDS-polyacrylamide gel and transferred onto polyvinylidene difluoride (PVDF) membranes (Millipore). Five percent of skim milk (BD) was used to block the non-specific binding for 1–2 h at 37°C, and then incubated with anti-LDHA (1:5000, Abcam), anti-VEGFA (1:10000, Abcam), and anti-PFKP (1:2000, Abcam) primary antibody at 4°C overnight. The membrane was washed with PBST buffer and then incubated with Goat Anti-Rabbit IgG (H + L) (1:10000, Jackson) for 2 h at 37°C. After washing with PBST buffer for six times, the membrane was exposed to enhanced chemiluminescence (ECL) reagents (Share-bio) and the Millipore ECL system, and then gray scanning was performed using TanonImage (4600, Tanon).

### Statistical Methods

In the current study, statistical analyses were performed using GraphPad prism (GraphPad software, San Diego, CA, United States). Continuous variables were presented with mean ± standard error of mean. Comparisons between groups were estimated using *t*-test, and *P* < 0.05 was considered statistically significant.

## Results

### CoCl_2_ Increased the Proliferation of HPASMC

**FIGURE 1:**
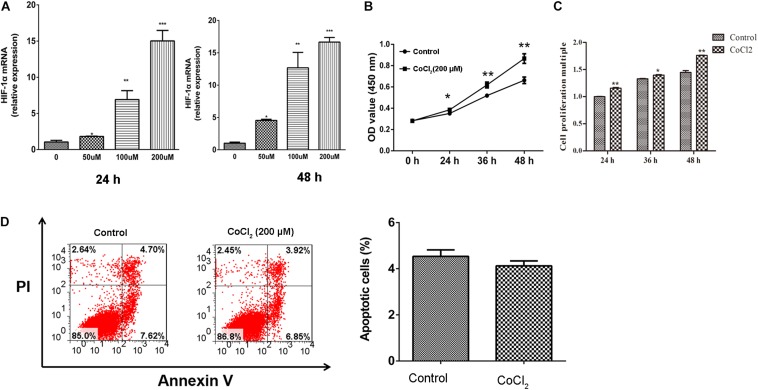
Effect of CoCl_2_ on HPASMCs. **(A)**, expression of HIF-1α in HPASMCs after being treated with CoCl_2_ at 24 h and 48 h; **(B)**, proliferation of HPASMCs after treatment with CoCl_2_ determined by CCK-8 assay; **(C)**, proliferation of HPASMCs after being treated with CoCl_2_ determined by CCK-8 assay BrdU assay, and **(D)**, apoptosis of HPASMCs after being treated with CoCl_2_. *P < 0.05 indicates that there is significantly different when compared with control groups. **P < 0.01 and ***P < 0.001.

After being treated with CoCl_2_, the expression of HIF-1α in HPASMCs was determined using RT-PCR, and the results showed that the expression levels of HIF-1α were significantly increased in HPASMCs with a dose-dependent manner at 24 and 48 h (Figure 1A). Then, 200 μM CoCl_2_ treatment for 48 h was utilized for the following investigation. Subsequently, the proliferation and apoptosis of HPASMCs were measured. The results showed that the proliferation of HPASMCs was significantly increased after treatment with 200 μM CoCl_2_ both determined by CCK-8 assay (Figure 1B) and by BrdU assay (Figure 1C), but no significant variation was identified in apoptosis after being treated with CoCl_2_, compared with the control group (Figure 1D).

### DEGs/lncRNA Screening and Functional Enrichment Analyses

**FIGURE 2:**
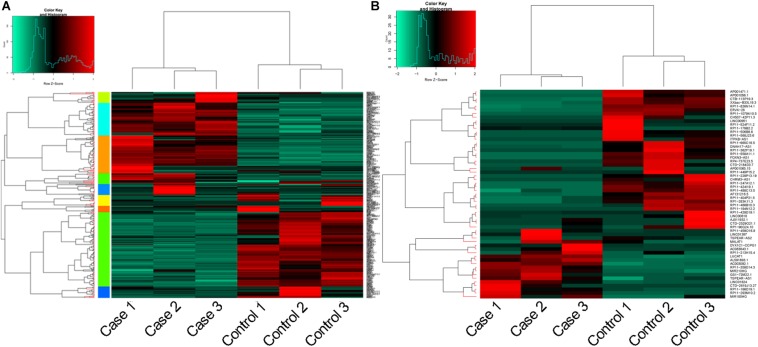
Heatmaps for DEGs and DELs. **(A)**, heatmap for DEGs; and **(B)**, heatmap for DELs. DEGs, differentially expressed genes; and DELs, differentially expressed long non-coding RNAs.

In total, 360 DEGs (178 up-regulated genes and 182 down-regulated genes) and 57 DELs (21 up-regulated lncRNAs and 36 down-regulated lncRNAs) were identified in CoCl_2_-treated HPASMCs compared with the controls. Heatmaps of DEGs and DELs are presented in Figures 2A,B. Moreover, GO enrichment analysis showed that the up-regulated DEGs were significantly enriched in the biological process (BP) terms of cellular response to hypoxia (*P* = 3.78 × 10^–09^), monosaccharide metabolic process (*P* = 4.61 × 10^–09^), cellular response to oxygen levels (*P* = 4.61 × 10^–09^), glycolytic process (*P* = 6.50 × 10^–09^), and pyruvate metabolic process (*P* = 8.32 × 10^–09^); and the down-regulated DEGs were closely associated with the BP terms of extracellular matrix organization (*P* = 2.42 × 10^–06^), collagen catabolic process (*P* = 3.30 × 10^–05^), multicellular organismal catabolic process (*P* = 3.79 × 10^–05^), extracellular matrix disassembly (*P* = 3.79 × 10^–05^), and single-substrate adhesion (*P* = 1.45 × 10^–04^) (Table 2). Meanwhile, KEGG enrichment analysis indicated that the up-regulated DEGs were primarily enriched in the pathways of Glycolysis/Gluconeogenesis (*P* = 6.98 × 10^–07^), Fructose and mannose metabolism (*P* = 2.16 × 10^–07^), and metabolic pathways (*P* = 2.28 × 10^–03^); the down-regulated DEGs played essential roles in the pathways of systemic lupus erythematosus (*P* = 1.06 × 10^–15^), ECM-receptor interaction (*P* = 3.82 × 10^–06^), Amoebiasis (*P* = 1.13 × 10^–05^), and focal adhesion (*P* = 4.91 × 10^–04^) (Table 2).

**TABLE 2:** Top 5 GO-BP terms enriched by the up- and down-regulated DEGs.

Terms	Count	*P* value		GeneSymbols
**A: Go functional enrichment**				
***Up-regulated DEGs***				
Cellular response to hypoxia	12	3.78 × 10^–09^	*NDRG1*, *EGLN3*, *FAM162A*, *HMOX1*, *PDK1*, *VEGFA*…	
Monosaccharide metabolic process	17	4.61 × 10^–09^	*ENO2*, *ALDOC*, *G6PD*, *GBE1*, *VEGFA*…	
Cellular response to oxygen levels	12	4.61 × 10^–09^	*NDRG1*, *EGLN3*, *FAM162A*, *HMOX1*, *PDK1 VEGFA*….	
Glycolytic process	10	6.50 × 10^–09^	*ENO2*, *ALDOC*, *HK2*, *LDHA*, *PFKFB3*…	
***Down-regulated DEGs***				
Extracellular matrix organization	14	2.42 × 10^–06^	*COL1A1*, *COL2A1*, *COL3A1*, *COL26A1*,*THBS1*.	
Extracellular structure organization	14	2.42 × 10^–06^	*COL1A1*, *COL2A1*, *COL3A1*, *COL26A1*,*THBS1*.	
Collagen catabolic process	7	3.30 × 10^–05^	*COL1A1*, *COL2A1*, *COL3A1*, *COL26A1*, *ADAMTS14*.	
Multicellular organismal catabolic process	7	3.79 × 10^–05^	*COL1A1*, *COL2A1*, *COL3A1*, *COL26A1*, *ADAMTS14*.	
Extracellular matrix disassembly	8	3.79 × 10^–05^	*COL1A1*, *COL2A1*, *COL3A1*, *COL26A1*, *LAMC2*.	

**B: KEGG functional enrichment**				

***Up-regulated DEGs***				
Glycolysis/Gluconeogenesis	8	6.98 × 10^–075^	*HKDC1*, *ALDOC*, *PGM1*, *HK2*, *ENO2*, *PFKP.*	
Fructose and mannose metabolism	6	2.16 × 10^–06^	*HKDC1*, *ALDOC*, *PFKFB4*, *HK2*, *PFKFB3*, *PFKP*	
Metabolic pathways	21	2.28 × 10^–03^	*HKDC1*, *GCNT3*, *TYRP1*, *GCLM*, *AK4*.	
***Down-regulated DEGs***				
Systemic lupus erythematosus	16	1.06 × 10^–15^	*HIST1H2AE*, *HIST1H3C*, *HIST2H3A*, *HIST2H4A*…	
ECM-receptor interaction	7	3.82 × 10^–06^	*LAMA4*, *THBS1*, *COL1A1*, *COL3A1*, *COL2A1*.	
Amoebiasis	7	1.13 × 10^–05^	*LAMA4*, *COL1A1*, *COL3A1*, *COL2A1*, *LAMC2*.	
Focal adhesion	7	4.91 × 10^–04^	*LAMA4*, *THBS1*, *COL1A1*, *COL3A1*, *COL2A1*.	

*GO, gene ontology; BP, biological process; DEG, differentially expressed genes.*

### Constructions of PPI Network and lncRNA-Target Network

**FIGURE 3:**
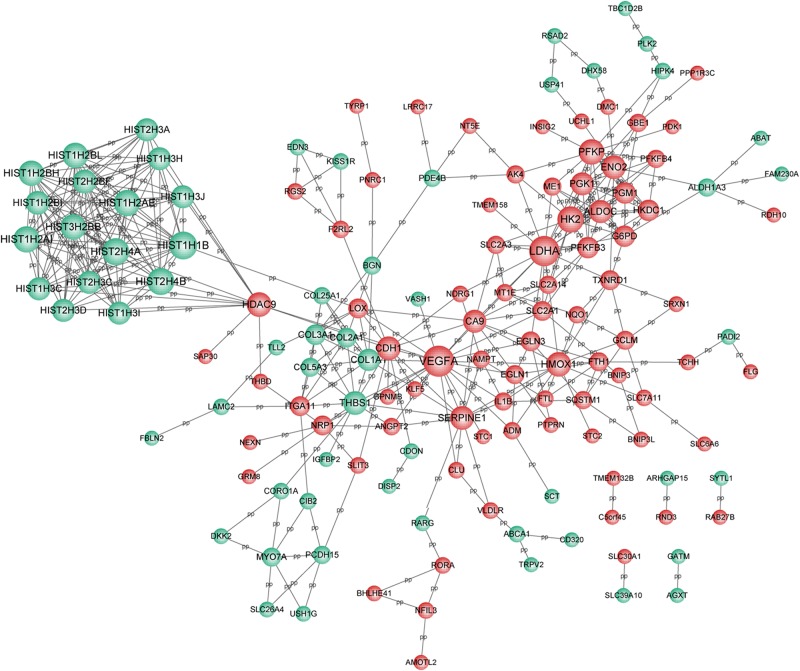
PPI network for DEGs. Red circle represents the up-regulated DEGs and green circle represents the down-regulated DEGs. Nodes size was positively correlated with node degree. PPI, protein-protein interaction network; and DEGs, differentially expressed genes.

Based on the selection criteria, PPI among DEGs encoded proteins were predicted and visualized using Cytoscape (Figure 3). The top 10 nodes involved in this network were: *VEGFA* (degree = 21), *LDHA* (degree = 20), *HIST1H1B* (degree = 17), *HIST2H4B* (degree = 16), *HIST2H4A* (degree = 16), *HK2* (degree = 15), *HIST1H2AI* (degree = 15), *HIST1H2AE* (degree = 15), *PFKP* (degree = 14), and *HIST3H2BB* (degree = 14).

**FIGURE 4:**
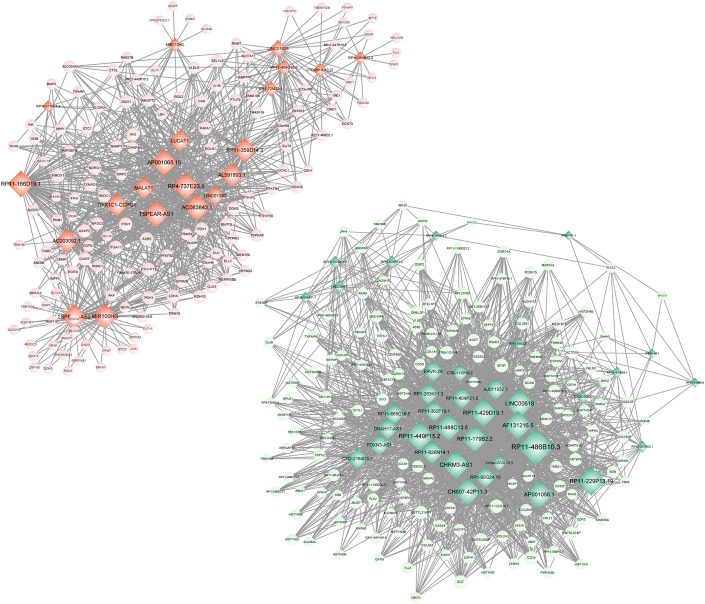
lncRNA-target network. Red circle represents the up-regulated DEGs, green circle represents the down-regulated DEGs, and diamond represents lncRNA. lncRNA, long non-coding RNA; and DEGs, differentially expressed genes.

Meanwhile, 286 targets by 57 lncRNAs were also selected from DEGs, and the lncRNA-target network was constructed with 343 nodes (proteins) and 2855 edges (PPI pairs) (Figure 4). The top 10 nodes with highest degrees were: *RP11-486B10.3* (degree = 127), *RP11-449P15.2* (degree = 97), *RP11-229P13.19* (degree = 93), *AF131216.5* (degree = 93), *LINC00618* (degree = 93), *RP11-429D19.*1 (degree = 93), *CHRM3-AS1* (degree = 93), *CH507-42P11.3* (degree = 88), *AP001056.1* (degree = 88), and *RP11-488C13.5* (degree = 85). Notably, *LDHA*, *PFKP*, and *VEGFA* as hub genes in PPI network presented a close correlation with miR-100HG and *TSPEA-AS2* in lncRNA-target network (Table 3).

**TABLE 3:** The interaction pairs of top 10 hub genes in PPI network and lncRNAs.

Tagrets	LncRNA	*P*-value
*VEGFA*	MIR100HG	0.017
	TSPEAR-AS2	0.017
*LDHA*	MIR100HG	0.017
	TSPEAR-AS2	0.017
	LINC01397	0.005
	TSPEAR-AS1	0.003
	AC083843.1	0.003
	DYX1C1-CCPG1	0.017
	AC003092.1	0.017
	AP001065.15	0.003
	MALAT1	0.017
	RP4-737E23.5	0.003
	AL591893.1	0.008
	LUCAT1	0.017
	RP11-359D14.3	0.008
	RP11-166D19.1	0.003
*PFKP*	MIR100HG	0.017
	TSPEAR-AS2	0.017
*HIST1H1B*	XXbac-B33L19.3	0.017
	CHRM3-AS1	0.005
	RP11-179B2.2	0.017
	RP11-449P15.2	0.003
	AP001056.1	0.008
	RP11-488C13.5	0.017
	CH507-42P11.3	0.008
	LINC00618	0.005
	RP11-826N14.1	0.000
	AF131216.5	0.005
	RP11-229P13.19	0.005
	RP11-486B10.3	0.008
	CTB-113P19.3	0.008
	RP11-429D19.1	0.005
*HIST2H4B*	FOXN3-AS1	0.005
	DNAH17-AS1	0.005
	RP11-404P21.5	0.008
	RP11-362F19.1	0.005
	RP11-179B2.2	0.017
	CTD-2184D3.7	0.005
	ERVK-28	0.000
	RP11-488C13.5	0.017
	AJ011932.1	0.000
	RP11-665C16.5	0.005
	RP1-90G24.10	0.008
	RP1-283K11.3	0.000
	RP11-486B10.3	0.008
*HIST2H4A*	FOXN3-AS1	0.005
	DNAH17-AS1	0.005
	RP11-404P21.5	0.008
	RP11-362F19.1	0.005
	RP11-179B2.2	0.017
	CTD-2184D3.7	0.005
	ERVK-28	0.000
	RP11-488C13.5	0.017
	AJ011932.1	0.000
	RP11-665C16.5	0.005
	RP1-90G24.10	0.008
	RP1-283K11.3	0.000
	RP11-486B10.3	0.008
*HK2*	LINC01397	0.005
	TSPEAR-AS1	0.017
	AC083843.1	0.017
	AP001065.15	0.017
	MIR210HG	0.017
	MALAT1	0.003
	RP4-737E23.5	0.017
	AL591893.1	0.008
	LUCAT1	0.003
	RP11-359D14.3	0.008
	RP11-166D19.1	0.017
*HIST1H2AI*	FOXN3-AS1	0.005
	DNAH17-AS1	0.005
	RP11-404P21.5	0.008
	RP11-362F19.1	0.005
	RP11-179B2.2	0.017
	CTD-2184D3.7	0.005
	ERVK-28	0.000
	RP11-488C13.5	0.017
	AJ011932.1	0.000
	RP11-665C16.5	0.005
	RP1-90G24.10	0.008
	RP1-283K11.3	0.000
	RP11-486B10.3	0.008
*HIST1H2AE*	FOXN3-AS1	0.005
	DNAH17-AS1	0.005
	RP11-362F19.1	0.005
	RP11-449P15.2	0.017
	CTD-2184D3.7	0.005
	RP11-665C16.5	0.005
	RP11-826N14.1	0.008
	RP11-486B10.3	0.008
*HIST3H2BB*	FOXN3-AS1	0.005
	DNAH17-AS1	0.005
	RP11-404P21.5	0.008
	RP11-362F19.1	0.005
	RP11-179B2.2	0.017
	CTD-2184D3.7	0.005
	ERVK-28	0.000
	RP11-488C13.5	0.017
	AJ011932.1	0.000
	RP11-665C16.5	0.005
	RP1-90G24.10	0.008
	RP1-283K11.3	0.000
	RP11-486B10.3	0.008

*PPI, protein-protein interaction.*

### Enrichment Analyses for Targets of lncRNA Isolated From DEGs

To further reveal the regulatory role of lncRNA, functional enrichment analyses for the targets of lncRNAs were also performed. The up-regulated targets were significantly enriched in the GO-BP terms of cell response to hypoxia (*P* = 9.60 × 10^–10^), cellular response to decreased oxygen levels (*P* = 9.60 × 10^–10^), cellular response to oxygen levels (*P* = 1.57 × 10^–09^), glycolytic process (*P* = 2.56 × 10^–09^), and pyruvate metabolic process (*P* = 2.84 × 10^–09^); the down-regulated targets were predominately associated with the GO-BP terms of extracellular matrix organization (*P* = 2.79 × 10^–06^), extracellular structure organization (2.79 × 10^–06^), collagen catabolic process (*P* = 3.56 × 10^–05^), multicellular organismal catabolic process (*P* = 4.13 × 10^–05^), and extracellular matrix disassembly (*P* = 4.13 × 10^–05^) (Table 4). The KEGG enrichment results showed that the up-regulated DEGs were significantly enriched in the pathways of glycolysis/gluconeogenesis (*P* = 1.85 × 10^–07^), fructose and mannose metabolism (*P* = 7.44 × 10^–07^), and metabolic pathways (*P* = 0.0019); the down-regulated targets were significantly enriched in the pathways of systemic lupus erythematosus (*P* = 1.06 × 10^–15^), ECM-receptor interaction (*P* = 3.82 × 10^–06^), amoebiasis (*P* = 1.13 × 10^–05^), and focal adhesion (*P* = 4.91 × 10^–04^) (Table 4).

**TABLE 4:** Functional enrichment analytical results for targets of lncRNAs.

Terms	Count	*P* value	Genes
**A: Go functional enrichment**			
***Up-regulated targets***			
Cellular response to hypoxia	12	9.60 × 10^–10^	*NDRG1*, *EGLN3*, *FAM162A*, *HMOX1*, *PDK1, VEGFA*.
Cellular response to decreased oxygen levels	12	9.60 × 10^–10^	*NDRG1*, *EGLN3*, *FAM162A*, *HMOX1*, *PDK1, VEGFA*.
Cellular response to oxygen levels	12	1.57 × 10^–09^	*NDRG1*, *EGLN3*, *FAM162A*, *HMOX1*, *PDK1, VEGFA*.
Glycolytic process	10	2.56 × 10^–09^	*ENO2*, *ALDOC*, *HK2*, *LDHA*, *PFKFB3, PFKP*,…
Pyruvate metabolic process	11	2.84 × 10^–09^	*ENO2*, *ALDOC*, *HK2*, *LDHA*, *PDK1, PFKP*.
***Down-regulated targets***			
Extracellular matrix organization	14	2.79 × 10^–06^	*COL1A1*, *COL2A1*, *COL3A1*, *COL26A1*, *THBS1*.
Extracellular structure organization	14	2.79 × 10^–06^	*COL1A1*, *COL2A1*, *COL3A1*, *COL26A1*, *THBS1*.
Collagen catabolic process	7	3.56 × 10^–05^	*COL1A1*, *COL2A1*, *COL3A1*, *COL26A1*, *ADAMTS14*.
Multicellular organismal catabolic process	7	4.13 × 10^–05^	*COL1A1*, *COL2A1*, *COL3A1*, *COL26A1*, *ADAMTS14*.
Extracellular matrix disassembly	8	4.13 × 10^–05^	*COL1A1*, *COL2A1*, *COL3A1*, *COL26A1*, *LAMC2*.

**B: KEGG functional enrichment**			

***Up-regulated targets***			
Glycolysis/Gluconeogenesis	8	1.85 × 10^–07^	*ALDOC*, *ENO2*, *HK2*, *HKDC1*, *LDHA*, *PFKP*.
Fructose and mannose metabolism	6	7.44 × 10^–07^	*ALDOC*, *HK2, HKDC1*, *PFKFB3*, *PFKFB4*, *PFKP,…*
Metabolic pathways	19	0.0019	*AK4*, *ALDOC, DGKD*, *DGKE*, *ENO2, PFKP*.
***Down-regulated targets***			
Systemic lupus erythematosus	16	1.06 × 10^–15^	*HIST1H2AE*, *HIST1H2AI*, *HIST1H2BH*, *HIST2H4A*,…
ECM-receptor interaction	7	3.82 × 10^–06^	*COL1A1*, *COL2A1, COL3A1*, *COL5A3*, *THBS1*,.
Amoebiasis	7	1.13 × 10^–05^	*CD14*, *COL1A1*, *COL2A1, COL3A1*, *COL5A3*, *LAMA4*.
Focal adhesion	7	4.91 × 10^–04^	*COL1A1*, *COL2A1*, *COL3A1*, *COL5A3*, *LAMA4, THBS1*.

*GO, gene ontology; BP, biological process; and KEGG, kyoto encyclopedia of genes and genomes.*

### The mRNA Expression of DEGs/DELs

**FIGURE 5:**
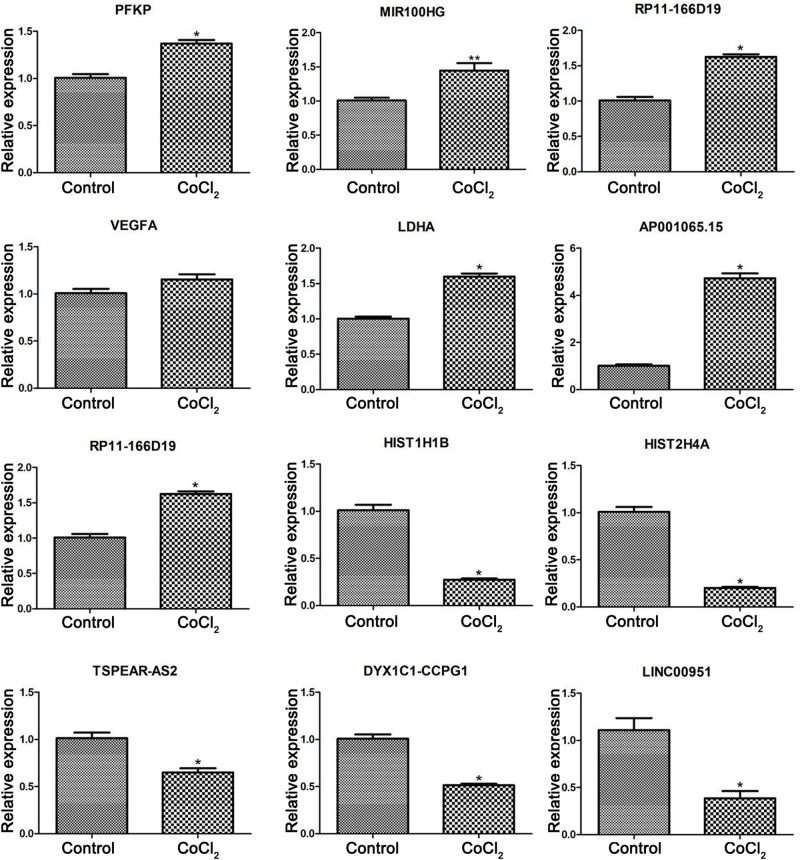
Validation of differentially expressed genes using real-time PCR. Compared with the control group, *P < 0.05, **P < 0.01.

According to the bio-informatics results, expression changes of several DEGs and DELs were validated using RT-PCR in HPASMCs. The validated results showed that expression levels of *PFKP*, miR-100HG, *RP11-166d19*, *VEGFA*, *LDHA*, and *AP001065.15* were significantly up-regulated, while expression levels of *RP11-568J2*, *HIST1H1B*, *HIST2H4A*, *TSPEAR-AS2*, *DYX1C1-CCPG*1, and *LINC00951* were significantly decreased in CoCl_2_-treated HPASMCs compared with the control groups, which were consistent with the findings based on the bioinformatics analyses (Figure 5).

### The Protein Expression of PFKP, VEGFA, LDHA

**FIGURE 6:**
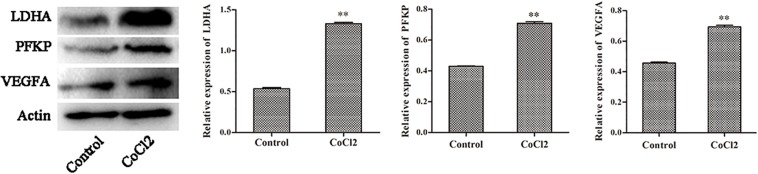
The protein expression of PFKP, VEGFA, and LDHA determined by western blotting. Compared with the control group, ^∗∗^*P* < 0.01.

The expression changes of *PFKP, VEGFA*, and *LDHA* at protein level were also determined by western blotting in HPASMCs (Figure 6). The results showed that the protein expression of *PFKP, VEGFA*, and *LDHA* were significantly increased in HPASMCs after COCl_2_ treatment. The results were consistent with the bioinformatics analyses.

## Discussion

Pulmonary arterial hypertension is considered a sub-population of PH, characterized by sustained pulmonary vascular resistance elevation that results in right heart failure and death (Pulido et al., 2013; Ball et al., 2014). Overwhelming evidence has demonstrated that chronic hypoxia serves as a potential trigger for pulmonary vascular remodeling, and *HIF-1*α is reported to play a critical role during this process (Ball et al., 2014). Although numerous achievements have been acquired in exploring the underlying pathogenesis of PAH, the corresponding molecular mechanisms have not been deciphered. In this study, CoCl_2_ could significantly increase the expression of *HIF-1*α and proliferation of HPASMCs and decrease the apoptosis of HPASMCs even though there was no statistical difference. Moreover, 360 DEGs (178 up-regulated and 185 down-regulated genes) and 57 DELs (21 up-regulated and 36 lncRNAs) were screened in HPASMCs treated with CoCl_2_ compared with the control group. Interestingly, we found that *LDHA*, *PFKP*, and *VEGFA*, which were regarded as hub genes in PPI network, exhibited a strong relationship with miR-100HG and TSPEAR-AS2 in lncRNA-target network. Additionally, the functional enrichment analysis of these three genes revealed that they probably played pivotal roles in the cellular response to hypoxia and the regulation of glycolysis.

*LDHA*, lactate dehydrogenase A (Cui et al., 2017) has been reported to be a crucial regulator in glycolytic metabolism. Two earlier studies illuminated that an energy shift from mitochondrial oxidative phosphorylation to glycolytic metabolism was strongly associated with the progression of PH, which basically induced cell hyper-proliferation and inhibited apoptosis (Koppenol et al., 2011; Cottrill and Chan, 2013). Consistently, our results implied that there was a significant increase for the proliferation of HPASMCs after 200 μM CoCl_2_ treatment but not in apoptosis. Later, Paulin and Michelakis (2014) elaborated the metabolic mechanisms of PAH and also emphasized that glycolysis was primarily responsible for the PAH occurrence and development. Zhang et al. (2018) argued that *LDHA* could dramatically enhance the activity of glycolysis in proliferating cells while *LDHA* knockdown retarded the glucose metabolism. In this work, we found that *LDHA* was significantly up-regulated in CoCl_2_-treated cells and remarkably enriched in glycolytic process, pyruvate metabolic process, and carbohydrate catabolic process. More notably, Zhang et al. (2014) investigated the expression of *LDHA* in monocrotaline-induced PHA rats and pointed out that *LDHA* was up-regulated, which was similar to our finding. Therefore, we speculated that *LDHA* might be involved in the PHA progression, presumably via mediating cell energy metabolism.

A huge amount of research has found that the vascular endothelial growth factor A (*VEGFA*) was closely correlated with the development of PH (Partovian et al., 2000; Papaioannou et al., 2009; Giannakoulas et al., 2014). Tiede et al. (2015) previously found that there was a higher level of *VEGFA* plasma compared with the controls based on the enzyme immunoassays, and stated that *VEGFA* could be regarded as a promising diagnostic maker for PH. Recently, Liu et al. (2018) revealed that the expression levels of *HIF-1*α and *VEGFA* were significantly elevated in human pulmonary arterial endothelia cells (HPAECs) with hypoxia and the activation of *HIF-1*α*/VEGFA* axis acted as a potential driver for vascular remodeling contributing to PH, which was consistent with our results that *HIF-1*α and *VEGFA* levels were dramatically increased in hypoxia-induced HPASMCs. Additionally, our functional enrichment analysis indicated that *VEGFA* played essential roles in cellular response to oxygen levels. Taken together, our results provided support for the hypothesis that the initiation of *HIF-1*α*/VEGFA* axis was implicated with HAP under hypoxia exposure.

Phosphofructokinase (*PFKP*) gene was involved with various cell activities and glucose metabolism by targeting to multiple cell receptors and was reported to be another prominent regulator in the progression of HP (Ameshima et al., 2003). Here, we found that this gene was up-regulated in HPASMCs and mainly participated in energy metabolism pathways such as fructose and mannose metabolism. Calvier et al. (2017) suggested that the expression of *PFKP* was increased in human PAH cells, which was in accordance with our finding. And they underscored that peroxisome proliferator-activated receptor gamma (PPARγ) could bind to *PFKP* to regulate the proliferation and metabolism of PH cells. Interestingly, *LDHA*, *PFKP*, and *VEGFA* all had a close correlation with miR-100HG and *TSPEAR-AS2* in our analysis. Unfortunately, few relevant investigations have been undertaken to explore the effect of the interaction between these genes and corresponding mRNAs on PAH occurrence and development. Therefore, the precise underlying mechanisms still need to be further elucidated.

In the current work, although we have identified several crucial gene signatures in PAH progression on the basis of bioinformatics methods, there are still limitations in our investigation. Firstly, the purity of HPASMCs should be determined before CoCl_2_ treatment to obtain more comprehensive results. Secondly, related animal experiments are required to confirm our findings even though we have verified significant genes by qPCR. Meanwhile, the exhaustive examinations containing a larger sample size was also needed, and the HPASMC RNA-seq data under different treatment should be further investigated. In addition, the detailed regulatory mechanisms associated with our confirmed genes remains to be deciphered.

## Conclusion

In conclusion, CoCl_2_-induced hypoxia could significantly increase *HIF-1*α level and the proliferation of HPASMCs but decrease the apoptosis. Meanwhile, the expression levels of *VEGFA*, *LDHA*, and *PFKP* were dramatically enhanced in hypoxia-induced HPASMCs, preferentially associated with glycolytic process and exhibited a close correlation with miR-100H*G* and *TSPEAR-AS*2, which were considered as pivotal players in PAH development. However, animal experimental verification and a comprehensive bioinformatics analysis will need to be performed in future.

## Data Availability Statement

The raw data was available at NCBI Sequence Read Archive (SRA) repository with Accession Number SRP081057.

## Author Contributions

TW: conception and design of the research, obtaining funding, and revision of manuscript for important intellectual content. HX: acquisition of data and analysis and interpretation of data. FH: statistical analysis. SC: drafting the manuscript. All authors read and approved the final manuscript.

## Conflict of Interest

The authors declare that the research was conducted in the absence of any commercial or financial relationships that could be construed as a potential conflict of interest.
